# Current and future on definitive concurrent chemoradiotherapy for inoperable locally advanced esophageal squamous cell carcinoma

**DOI:** 10.3389/fonc.2024.1303068

**Published:** 2024-01-26

**Authors:** Renxian Xie, Qingxin Cai, Tong Chen, Hongxin Huang, Chuangzhen Chen

**Affiliations:** ^1^ Department of Radiation Oncology, Cancer Hospital of Shantou University Medical College, Shantou, China; ^2^ Shantou University Medical College, Shantou, China

**Keywords:** esophageal squamous cell carcinoma, concurrent chemoradiotherapy, radiotherapy, chemotherapy, immunotherapy

## Abstract

Esophageal squamous cell carcinoma (ESCC) is an aggressive and fatal disease that is usually diagnosed when the chances for surgical intervention has been missed. Definitive concurrent chemoradiotherapy (dCRT) is the first choice of treatment for inoperable locally advanced esophageal squamous cell carcinoma (LA-ESCC). Nevertheless, the local recurrence rate for esophageal cancer patients undergoing dCRT remains high at 40-60%, with a 5-year overall survival rate of solely 10-30%. Immunotherapy in combination with dCRT is a promising treatment for inoperable LA-ESCC, for that improved long-term survival is expected. The present review provides a comprehensive overview of the evolutionary trajectory of dCRT for LA-ESCC, delineates notable relevant clinical studies, addresses unresolved concerns regarding the combination of dCRT with immunotherapy, and highlights promising directions for future research. When dCRT is combined with immunotherapy, the following aspects should be carefully explored in the future studies, including the optimal irradiation dose, segmentation scheme, radiotherapy technique, timing, sequence and duration of radiotherapy, and the selection of chemotherapeutic and immunologic drugs. In addition, further investigations on the mechanisms of how dCRT combined with immunotherapy exerts synergistic anti-tumor effects and molecular biomarkers ensuring precise screening of ESCC patients are needed.

## 1 Introduction

Esophageal cancer (EC) is a highly aggressive and fatal disease, which ranks the 7th in incidence and the 6th in mortality among all cancers in the world (1). According to the Global Cancer Statistics 2020, there were 604,000 new cases of esophageal cancer, meanwhile, 544,000 people died of this disease (1). EC can be classified into esophageal squamous cell carcinoma (ESCC) and esophageal adenocarcinoma according to histotype, and they display distinct differences in pathogenesis and biological characteristics, which lead to diverse treatment approaches and prognosis. For ESCC in early stages, surgery is the primary option. However, patients come to the clinic are frequently diagnosed as locally advanced esophageal squamous cell carcinoma (LA-ESCC), losing the chance for radical resection (2). Inoperable LA-ESCC is, by definition, refers to locally advanced tumors or regional lymph node metastases, without distant metastases (ie, American Joint Committee on Cancer stage ≥ T2 or N+, M0), in these cases, radical surgery is null or declined (3). Patients with inoperable LA-ESCC have a poor prognosis and the mortality is high (4, 5). For this group of patients, National Comprehensive Cancer Network (NCCN) guidelines recommend definitive concurrent chemoradiotherapy (dCRT) as the standard of care (6). However, dCRT has a few limitations: EC patients receiving dCRT have a high local recurrence rate up to 40-60% (7), with a 5-year overall survival rate of solely 10-30% (8). Researchers have been committed to exploring treatment modalities that can improve the outcomes of patients with LA-ESCC. The present review provides a comprehensive overview of the evolutionary trajectory of dCRT for LA-ESCC, delineates notable relevant clinical studies, and highlights promising directions for future research.

## 2 Evolution of dCRT for inoperable LA-ESCC

The combination of chemotherapy and radiotherapy was introduced in the 1970s with the goal of improving treatment outcomes by combining the two treatments to more effectively kill tumor cells. Subsequently, numerous clinical trials were initiated to investigate the efficacy of dCRT for the treatment of various cancers, including esophageal cancer. These trials demonstrated that dCRT significantly improved the survival and prognosis of patients with tumors compared to radiotherapy or chemotherapy alone. Moreover, the development of more potent chemotherapeutic agents and advanced radiotherapy techniques has further increased the potential of dCRT. As clinical trials continue to show promising results, dCRT is gradually being recognized as the standard treatment for inoperable LA-ESCC.

Currently, dCRT stands as the recommended standard of care for inoperable LA-ESCC, which is derived from the results of the prospective study RTOG 85-01. Prior to RTOG 85-01, several randomized controlled studies had compared the efficacy of dCRT versus radiotherapy alone in patients with inoperable LA-ESCC. However, the results of these studies are inconclusive, which may be due to insufficient sample sizes in some studies and the use of different chemotherapy regimens in different studies (9–12). RTOG 85-01 was a multicenter, randomized controlled phase III clinical trial that demonstrated dCRT significantly improves overall survival (OS) in patients with locally advanced esophageal cancer compared to radiotherapy alone (13). This study revealed that the 5-year OS rate of dCRT for locally advanced esophageal cancer is 26%, compared to 0% of radiotherapy alone. This result established the essential foundational and ushered in the era of dCRT for inoperable LA-ESCC. Other therapies such as neoadjuvant chemotherapy plus dCRT and dCRT plus adjuvant chemotherapy, have since then been investigated for their efficacy and safety compared to dCRT in patients with inoperable LA-ESCC, hoping to boost the therapeutic efficacy of dCRT. Xi M et al. reported that neoadjuvant chemotherapy (docetaxel and cisplatin) combined with dCRT in patients with inoperable LA-ESCC does not significantly improve patients’ 5-year OS and progression-free survival (PFS) compared to dCRT alone (14). The 5-year OS in the two groups was 31.8% versus 29.1% and the PFS was 30.5% versus 25.5%, respectively, with no significant statistical difference between these two groups. Similarly, another study conducted by Chen MQ et al. indicated no significant differences in OS, local failure-free survival and distal failure-free survival between the patients treated by neoadjuvant chemotherapy combined with dCRT or by dCRT alone (15). What’s more, a retrospective study including 244 patients with inoperable LA-ESCC showed no statistically significant difference was uncovered in the OS and the PFS between patients treated by dCRT combined with adjuvant chemotherapy and those by dCRT alone (16). In conclusion, neither neoadjuvant chemotherapy combined with dCRT nor dCRT combined with adjuvant chemotherapy appears to significantly improve therapeutic outcomes for LA-ESCC patients and cannot shake the essential status of dCRT in treating LA-ESCC. Currently, dCRT remains the standard of care for inoperable LA-ESCC. However, the specific regimen of dCRT remains somewhat controversial. To date, there are several prospective clinical trials are ongoing and will help to determine the specific regimen of dCRT for patients with LA-ESCC (**Table 1**). Nevertheless, it should be noted that immunotherapy plays an increasingly important role in comprehensive cancer therapy and dCRT combined with immunotherapy for LA-ESCC is a promising direction for future research.

**Table 1:** Specific regimens for definitive concurrent chemoradiotherapy for unresectable esophageal squamous cell carcinoma: ongoing trials.

Registration	Phase	Participants	Population	Study Cohort	Control Cohort	Primary Endpoints	Secondary Endpoints
NCT04278287	I/II	105	Pathological T3N1M0-1b and T4N0-1M0-1b ESCC(M1b limited to clavicular or celiac lymph node metastasis)	Radiotherapy (59.92 Gy/28F) + Chemotherapy (Albumin-Bound Paclitaxel + Cisplatin)		LCR	AEs, ORR, DFS, PFS, OS
NCT04115618 2017-ESCCSIB	II	65	Stage I-IVA ESCC (AJCC 6th,2009)	Chemotherapy (Docetaxel + Cisplatin) + Radiotherapy(63 Gy/28F)		AEs	PFS, OS
NCT03790553 ESO-Shanghai 12	III	646	T1N1-3M0, T2-4NxM0, TxNxM1 ESCC (supraclavicular lymph node metastasis only)(AJCC 8th)	Chemotherapy (NP) + Radiotherapy (50.4Gy/28F, involved field irradiation)	Chemotherapy (NP) + Radiotherapy (61.2Gy/34F, involved field irradiation)	OS	LCR, PFS, QoL

T, Tumor; N, regional lymph node; M, Metastasis; ESCC, esophageal squamous cell carcinoma; AJCC, American Joint Committee on Cancer; NR, not reported; F, fraction; LCR, Local control rate; AEs, adverse events; ORR, objective response rate; DFS, disease-free survival; PFS, progression-free survival; OS, overall survival; QoL, quality of life.

## 3 Dose of radiotherapy

Routine split irradiation with a split dose of 1.8-2.0 Gy is recommended for LA-ESCC patients undergoing dCRT. Researchers have been persistently exploring other modes of radiotherapy segmentation, such as hypofractionated radiotherapy, accelerated or hyperfractionated radiotherapy, late course accelerated hyperfractionated radiotherapy (LCAF), etc. The study conducted by Zhou et al. (17) included 58 patients with inoperable stage II-IVB LA-ESCC undergoing moderately hypofractionated radiotherapy (60Gy/24 fractions) with S-1 synchronized chemotherapy. These patients reached an objective response rate of 91.3% and a complete response rate (CRR) of 60.3%, as five of them (8.6%) developed grade ≥ 3 esophagitis. However, the treatment tolerance rate was only 78.8% when the fractionated dose of from 3.0 to 5.0 Gy, with further patients (21.2%) experiencing grade ≥ 3 acute radiation toxicity (18). A meta-analysis including 20 randomized controlled clinical studies showed that accelerated or hyperfractionated radiotherapy significantly improves response rates, OS, and local control rates in patients with ESCC, as it also increases the risk of acute radiation reactions (19). Better results have been achieved by LCAF. The results of a randomized controlled clinical study conducted by Shi et al. (20) demonstrated that the 5-year actuarial survival rate and disease-free survival rate of LCAF group were 34% and 42%, respectively, versus 15% and 15% for 5-year actuarial survival rate and disease-free survival rate respectively in conventional fractionation group. The LCAF group exhibits a superior local control rate (55%) compared with the conventional fractionation group (21%). Moreover, patients in the LCAF group exhibited a tolerable acute radiation response, and no increase in late radiation responses was observed in patients in the LCAF group compared with those in conventional fractionation group at 5 years. Nevertheless, the development of LCAF still faces a range of challenges, including not only an increased workload for radiotherapists but also an elevated risk of positioning errors, thereby impacting the repeatability and accuracy of radiotherapy and limiting the clinical utilization of LCAF. Overall, moderately hypofractionated radiotherapy (the fractionated dose of from 2.0 to 3.0 Gy) seems worth a try, but we need more clinical studies to validate it.

The dose of radical radiotherapy for LA-ESCC has been controversial. In Europe and the United States, the standardized total dose of dCRT is 50-50.4 Gy (6), while 60-70 Gy is most frequently used in Asian countries (21, 22). According to the Lancet Seminar on esophageal carcinoma, EC patients receiving dCRT had a local recurrence rate of 40-60%, which is apparently not optimistic (7). It was then explored whether increasing the dose of radiotherapy could be a possible solution to lower local recurrence rate. However, no significant benefit in terms of locoregional control or survival was found in the RTOG9405 study when comparing a high dose (64.8 Gy) to the standard dose (50.4 Gy) of dCRT for EC (23). Among 218 eligible patients, there were no significant differences between the high-dose and standard-dose groups in terms of median survival (13.0 months vs 18.1 months), 2-year OS rate (31% vs 40%), and local/regional failure rate (56% vs 52%). This results in the maintenance of agreed norm for dCRT dosage ranging from 50-50.4 Gy in the majority of Western countries.

Unlike in Western countries, ESCC is the predominant pathologic type in Asian countries and has better survival and local control rates than adenocarcinoma after dCRT (24, 25). Moreover, both RTOG 85-01 and RTOG 94-05 used conventional radiotherapy techniques rather than intensity-modulated radiotherapy(IMRT) or volume-modulated arc therapy(VMRT) techniques that can further reduce the dose of exposure to surrounding normal tissue. The results of RTOG 94-05 were somewhat biased by the severe toxicity and low completion rate of dCRT using conventional radiotherapy techniques. Considering these factors, higher radiation doses (60-70 Gy) using modern radiotherapy techniques such as IMRT or VMRT are more common in clinical practice in Asian countries (26). Several retrospective and prospective studies compared the standard dose (50 Gy) with high doses (≥60 Gy) for EC in the last few decades, but these studies did not reach a consistent conclusion on whether high doses improve local control and survival rate without increasing therapeutic toxicity (27–31). Subsequently, several prospective multicenter randomized controlled studies based on modern radiotherapy technology compared the efficacy and safety of high-dose and the standard dose radiotherapy in patients with inoperable locally advanced esophageal cancer. The ARTDECO study showed no significant difference in 3-year local progression-free survival (73% vs 70%) and 3-year locoregional progression-free survival (59% vs 52%) between the high-dose radiotherapy group and the standard-dose radiotherapy group (32). Similarly, another study comparing the efficacy and safety of 60 Gy with 50 Gy in individuals with LA-ESCC showed no significant difference in 3-year locoregional progression-free survival (49.5% vs 48.4%), 3-year OS rate (53.1% vs 52.7%), and 3-year PFS rate (46.4% vs 46.1%). Moreover, more grade 3 radiation pneumonitis occurred in the 60 Gy group compared with the 50 Gy group (33). Additionally, the study by You et al. showed that the high dose of 59.4 Gy did not significantly improve the median OS of patients with LA-ESCC, with median OS of 28.1 and 26.0 months in the 59.4Gy and 50Gy groups, respectively (34). In conclusion, the results of these multicenter prospective clinical studies led to the same result: patients with locally advanced esophageal cancer receiving high radiotherapy do not achieve a more ideal OS or PFS, similar to the result of the RTOG9405 study.

It is worth noting that elective nodal irradiation (ENI) was used as the radiotherapy target area in all the clinical studies mentioned above. Increasing amounts of studies are now showing that lymphocytes (especially T-lymphocytes) play an essential role in the prognosis of patients with tumors, and lymphocytopenia could lead to worse prognosis. ENI irradiates a bunch of lymph node regions, which may aggravate radiotherapy side effects and deteriorate prognosis for patients, and this may be the reason for invalid beneficial effect from high-dose radiotherapy. Unlike previous prospective studies comparing high-dose to standard dose, the ESO-Shanghai 12 study (35) not only evaluated the prognosis of patients based on PET-CT, but also used involved field irradiation (IFI), which is noteworthy.

## 4 Range of radiotherapy targets

The range of clinical target volume (CTV) of radical radiotherapy for EC patients has not been uniform. In the beginning, CTV asked for an extension of 3-5 cm above and below the gross tumor volume (GTV) and 2 centimeters around the GTV. In comparison, a study by Gao et al. (36) found that approximately 94% of patients with ESCC had microscopic tumor extension of less than 3 cm. Tsutsui et al. (37) found that 94% of proximal microscopic tumor extension in patients with ESCC were located within 3 cm of the tumor margins, and 83% of distal microscopic tumor extension were located within 3 cm of the tumor margins, which was similar to that of Gao et al. They also found that the maximum distance of subepithelial spread of the tumor in patients with ESCC was 106 mm, and the maximum distance of spread along lymphatic or vascular vessels was 79 mm. Button et al. (38) analyzed the pattern of recurrence in patients with EC treated with dCRT. In their study, the CTV was GTV plus 3 cm of superior/inferior dilatation and 1 cm of peripheral dilatation. Recurrence was experienced by 88 out of 145 patients with a median follow-up of 18 months. The important thing to keep in mind is that 96% of localized recurrences occurred within the radiation field, which means expanding CTVs cannot reduce the localized recurrence rate of patients. Therefore, NCCN guidelines recommend that the CTV includes the GTV plus 3 to 4 cm of extension above and below esophagus and cardia and 1 cm of extension around it.

The use of IFI or ENI for the extent of radiotherapy target areas in dCRT for EC has been controversial. ENI is recommended for dCRT for LA-ESCC according to NCCN guidelines (6). However, a growing body of evidence supports the choice of IFI for dCRT in patients with EC. The results of a multicenter prospective clinical study comparing the effectiveness of ENI and IFI in patients with LA-ESCC undergoing dCRT revealed that there is no notable distinction between the ENI and IFI groups in terms of median PFS (20.3 months vs 21.4 months), OS (32.5 months vs. 34.9 months), local recurrence-free survival (25.0 months vs. 26.6 months), 5-year OS rate (29.8% vs 30.7%) and 5-year PFS rate (26.9% vs 27.7%) (39). Besides, Zhao et al. found no increase in tumor treatment failure among LA-ESCC patients who received IFI irradiation compared to those who received ENI irradiation (40). The incidence of field recurrence was only 2% in patients treated with IFI (41). Although patients irradiated with ENI and IFI had similar efficacy and field failure rates, the relatively smaller irradiation field of IFI helped to protect patients’ normal tissues and reduce treatment side effects.

More importantly, more and more studies have shown that lymphocytes (especially T-lymphocytes) play a very important role in the immunotherapy of tumor patients, and that lymphopenia has a negative impact on prognosis during radiotherapy (42, 43). T-lymphocytes are an indispensable part of the immune system and contribute to the fight against tumors in multiple ways. Firstly, they recognize and bind to tumor cell surface molecules, activating cytotoxic T cells that then eliminate the tumor cells (44). Secondly, T-lymphocytes secrete cytokines such as interferon-γ (IFN-γ) and interleukin-2 (IL-2), which enhance the immune system’s ability to surveil and attack tumor cells (45). Furthermore, these cells generate immunological memory cells that rapidly activate when tumor cells reappear, improving the efficacy of the immune response against tumors. Additionally, T-lymphocytes regulate the immune microenvironment, influencing tumor growth and metastasis (46). Immunotherapeutic strategies targeting T-lymphocytes, such as T cell receptor (TCR) gene engineering and cytokine therapy, can further strengthen the immune response against tumor cells (47). Therefore, in tumor treatment, it is essential to fully mobilize and protect the function of T-lymphocytes to enhance the effectiveness of anti-tumor therapy (46). In summary, IFIs are increasingly being selected in order to preserve lymphocyte function and minimize lymph node irradiation. More clinical studies should be conducted based on IFIs.

## 5 Radiotherapy technology

Over the past few decades, advances in radiation technology including IMRT, VMRT, cone-beam computed tomography, proton therapy, and better patient immobilization have radically improved outcomes of patients with EC. IMRT has been widely used in the treatment of EC, and it has better target area conformability than 3-Dimensional Conformal Radiotherapy (3D-CRT), which can reduce the exposure of vital organs such as heart and lung (48). Despite the fact that a retrospective study found that IMRT technology and 3D-CRT technology did not have a significant difference on 3-year OS (54.4% vs 49.6%) and progression-free survival (42.8% vs 45.8%) in patients with cervical cancer (49), more retrospective studies have found that IMRT improves local control and patient survival in EC patients compared to 3D-CRT. The study by Bai et al. found significantly better 5-year OS (42.8%) in patients who received IMRT compared to 5-year OS (35.4%) in patients who received 3D-CRT (50). Similarly, Lin et al. found that patients with LA-ESCC who underwent IMRT-based dCRT had a higher survival rate (51). Moreover, patients who underwent 3D-CRT had a significantly increased risk of death (72.6% vs 52.9%) and local recurrence compared with IMRT (52). Based on these results, 3D-CRT has been replaced by IMRT in the treatment of EC.

Some retrospective studies have shown that proton radiotherapy significantly reduces lung and cardiac exposure compared to IMRT, which can further reduce the incidence of long-term cardiac injuries as well as radiation pneumonitis in EC patients (53–55). In addition, a phase II prospective randomized controlled study found that proton radiotherapy reduces the incidence and severity of adverse events in patients with locally advanced EC compared with IMRT, and that PFS is not shortened as a result (56). Another retrospective study has similarly demonstrated that IMRT has worse OS (hazard ratio, 1.454; P = 0.01), PFS (hazard ratio, 1.562; P = 0.001), and LRFFS (hazard ratio, 1.461; P = 0.041) than proton radiotherapy (57). However, proton radiotherapy technology is not widely used in the clinic because of its high cost, which has prevented large-scale clinical validation of the efficacy and safety of proton therapy compared with IMRT in patients with EC.

Therefore, IMRT is recommended for patients with EC who need to undergo radical radiotherapy, which can not only ensure patients’ efficacy, but also effectively protect normal tissues.

## 6 Chemotherapeutic regimen

The status of dCRT in LA-ESCC was established in the RTOG 8501 (13) and INT0123 study (23), where the concurrent chemotherapy regimen of cisplatin combined with fluorouracil was applied. Since then, this regimen has been used as a standard control in clinical trials of dCRT for EC. However, cisplatin combined with fluorouracil has significant toxic side effects and the efficacy is still unsatisfactory. Therefore, researchers have been exploring concurrent chemotherapeutic regimens that are both radiosensitizing and low-toxicity, with the expectation that they will improve the outcomes of patients treated non-surgically for locally advanced EC.

In recent years, it has been found in several studies that the combination of oxaliplatin not only shows non-inferiority to cisplatin in terms of efficacy, but is also more convenient for clinical use. The PRODIGE5/ACCORD17 study found no significant difference in median PFS (9.7 months vs. 9.4 months) or in the incidence of grade 3 or 4 adverse events between patients receiving the FOLFOX regimen (Leucovorin Calcium + Fluorouracil + Oxaliplatin) and those receiving fluorouracil combined with cisplatin regimen (58). And the FOLFOX regimen is more convenient than the fluorouracil combined with cisplatin regimen because it takes less time to complete chemotherapy and can be administered on an outpatient basis. The 2023 NCCN guidelines (6) recommend combining oxaliplatin in dCRT more than cisplatin. Therefore, oxaliplatin combined with fluorouracil is the standard chemotherapy regimen of dCRT for EC (58, 59), and paclitaxel combined with carboplatin (60), fluorouracil combined with cisplatin (23), cisplatin combined with docetaxel or paclitaxel (61–63), paclitaxel combined with fluoropyrimidine (fluorouracil or capecitabine) (64) and FOLFOX regimen are optional concurrent chemotherapy regimens. The toxicity and side effects of different chemotherapy regimens vary greatly, so clinicians should make the choice according to the actual situation of patients in clinical practice. For example, the side effects of the paclitaxel combined with fluorouracil regimen and the cisplatin combined with fluorouracil regimen were distinctly different, with a higher incidence of early grade 3 and higher leukocyte decline, radioskin lesions, and radiation pneumonitis in the paclitaxel combined with fluorouracil regimen, and a higher incidence of early side effects such as grade 3 and higher nausea, vomiting, anorexia, fatigue, anemia, and thrombocytopenia in the cisplatin combined with fluorouracil regimen. Besides, cisplatin, a first-generation platinum drug, has more significant nephrotoxicity, gastrointestinal reactions, and neurotoxicity, whereas the third-generation platinum drug oxaliplatin has lower toxicity compared to cisplatin, with neurotoxicity as its main side effects.

## 7 Development of therapeutic strategies for inoperable LA-ESCC in the immune era

After the KEYNOTE-001 study, which showed superior efficacy of immunotherapy in patients with prior radiotherapy, researchers have investigated the effectiveness of combining radiotherapy with immunotherapy in EC patients (65). Despite the surprising results of immunotherapy in the treatment of advanced EC patients (66–68), the study of immunotherapy combined with dCRT in inoperable LA-ESCC is still in the exploratory stage.

In 2021, a single-arm, single-center, open-label, phase Ib clinical study of camrelizumab in combination with dCRT for the treatment of inoperable LA-ESCC showed that dCRT plus anti-PD-1 antibody is safe and feasible (69). In this study, 20 patients were included, with 2 patients showing complete response, 13 patients showing partial response, and 3 patients having stable disease status. Forty cases of inoperable LA-ESCC were included in the EPOC1802 study, a prospective, multicenter, single-arm clinical study (70). The results of its phase II study indicated that patients with inoperable LA-ESCC who underwent dCRT (cisplatin, 5-FU combined with radiotherapy) followed by atilizumab maintenance therapy for 1 year had a CRR of up to 42.1% and an objective response rate of up to 65.8%. And this study did not identify any new safety concern. Results from another prospective, single-arm, phase II clinical study suggested that the combination of durvalumab and tremelimumab with dCRT for the treatment of patients with inoperable LA-ESCC resulted in significantly prolonged PFS and OS when compared to patients who had previously received dCRT alone (71). This study included 40 patients with a median follow-up of 27.5 months, and these patients exhibited a 2-year PFS rate of 57.5% and an OS rate of 75%. What’s even more surprising is that this study documented the most minimal rate of failure in the field (17.5%) compared to all other published articles. However, it is worth noting that eight patients discontinued treatment because of adverse events, seven of whom permanently discontinued consolidation immunotherapy owing to immune-mediated adverse events within a few months of initiating treatment. The types of adverse events included colitis, adrenocortical insufficiency, and pneumonia. The EC-CRT-001 study also recently reported results from its Phase II clinical study. In patients with LA-ESCC, the combination of toliparibumab with definitive dCRT improves patient outcomes with tolerable toxicities (72). 26 of 42 patients achieved complete response with a median duration of response of 12.1 months. The 1-year OS rate for these patients was 78.4% and the 1-year PFS rate was 54.5%. Overall, these four clinical studies provide preliminary evidence that patients with LA-ESCC undergoing dCRT combined with immunotherapy have a good response rate and that the side effects are tolerable.

However, due to the small sample size included in these studies, we need evidence from larger phase III randomized controlled clinical studies to validate the safety and efficacy of dCRT combined with immunotherapy compared with dCRT alone in patients with inoperable LA-ESCC.

Although no such phase III clinical study data have been published, there are many phase III randomized multicenter trials of immunotherapy in combination with dCRT in progress (**Table 2**). In addition, there are several clinical studies exploring whether immunotherapy followed by sequential dCRT (73, 74) or dCRT followed by sequential immunotherapy (75) can improve patient outcomes. We need to pay attention to these studies to determine whether immunotherapy combined with dCRT can further improve outcomes.

**Table 2:** Definitive concurrent chemoradiotherapy combined with immunotherapy for unresectable esophageal squamous cell carcinoma: phase III ongoing trials.

Registration	Phase	Participants	Population	Study Cohort	Control Cohort	Primary Endpoints	Secondary Endpoints
NCT03957590 RATIONALE311	III	370	Locally advanced ESCC	Tislelizumab + Chemotherapy (Paclitaxel + Cisplatin) + Radiotherapy(50.4Gy/28F)	Placebo + Chemotherapy (Paclitaxel + Cisplatin) + Radiotherapy(50.4GY/28F)	PFS	ORR, DoR, OS, AEs
NCT04210115 KEYNOTE-975	III	700	TxN+M0,T2-T4aNxM0 ESCC/EAC/GEJC or TxN+M1(cervical or upper thoracic esophageal carcinoma with supraclavicular lymph node metastases only)	Pembrolizumab + Chemotherapy (Cisplatin + 5-FU or FOLFOX) + Radiotherapy(50/25F or 60Gy/30F)	Placebo + Chemotherapy (Cisplatin + 5-FU or FOLFOX) + Radiotherapy(50/25F or 60GY/30F)	OS, EFS	AEs
NCT04550260 KUNLUN	III	600	Stage II-IVA ESCC	Durvalumab + Chemotherapy (Cisplatin + 5-FU or Cisplatin + Capecitabine) + Radiotherapy(50-64Gy)	Placebo + Chemotherapy (Cisplatin + 5-FU or Cisplatin + Capecitabine) + Radiotherapy(50-64GY)	PFS	OS, AEs
NCT04426955 ESCORT-CRT	III	396	Locally advanced ESCC	Camrelizumab + Chemotherapy (Paclitaxel + Cisplatin) + Radiotherapy(50.4Gy/28F)	Placebo + Chemotherapy (Paclitaxel + Cisplatin) + Radiotherapy(50.4GY/28F)	PFS	OS, ORR, DoR, AEs
NCT04543617 SKYSCRAPER-07	III	750	Unresectable ESCC	Chemotherapy (Platinum-based) + Radiotherapy(standard care dose) + Tiragolumab + Atezolizumab (Cohort A) Chemotherapy (Platinum-based) + Radiotherapy(standard care dose) + Tiragolumab Placebo+ Atezolizumab (Cohort B)	Chemotherapy (Platinum-based) + Radiotherapy(standard care dose) + Tiragolumab Placebo + Atezolizumab Placebo	PFS, OS	ORR, DoR, QoL, AEs
NCT04821778	III	2000	Stage I-IVa ESCC/EAC(AJCC 8th)	Anti-PD-1/PD-L1 antibody + Chemotherapy (Paclitaxel/Platinum/5-FU) + Radiotherapy(50-66Gy/25-30F)	Chemotherapy (Paclitaxel/Platinum/5-FU) + Radiotherapy(50-66GY/25-30F)	OS	PFS, AE, LRFS, DMFS
NCT04404491 ITCRTECR	III	240	Stage II-IVA ESCC	Camrelizumab + Chemotherapy (Oxaliplatin +Capecitabine) + Radiotherapy(50-50.4Gy/25-28F)	Placebo + Chemotherapy (Oxaliplatin +Capecitabine) + Radiotherapy(50-50.4GY/25-28F)	AEs, PFS	ORR, OS
NCT05919030	III	155	unresectable advanced, recurrent or metastatic ESCC(AJCC 8th)	Tislelizumab + Chemotherapy (Nab paclitaxel + Cisplatin)+Radiotherapy(39.6Gy/18F)	Tislelizumab + Chemotherapy (Nab paclitaxel + Cisplatin)	PFS	OS, ORR, DOR, AEs

T, Tumor; N, regional lymph node; M, Metastasis; ESCC, esophageal squamous cell carcinoma; EAC, esophageal adenocarcinoma; GEJC, gastroesophageal junction cancer; AJCC, American Joint Committee on Cancer; 5-FU, 5-fluorouracil; FOLFOX, Leucovorin Calcium + Fluorouracil + Oxaliplatin; F, fraction; PFS, progression-free survival; DOR, duration of objective response; ORR, objective response rate; EFS, event-free survival; LRFS, locoregional recurrence free survival; OS, overall survival; AEs, adverse events; CCR, clinical complete response; PFS, progression-free survival; DMFS, distant metastasis free survival; QoL, quality of life.

## 8 Future directions

### 8.1 Definitive concurrent chemoradiotherapy combined with immunotherapy

Immunotherapy combined with dCRT has been proven effective and safe in some preliminary clinical studies, which has led to more and more clinical studies exploring dCRT combined with immunotherapy gradually. However, when dCRT is combined with immunotherapy, the optimal chemotherapy regimen, the optimal fractionation regimen, target volume and dosage of radiotherapy and the optimal timing of the combination of immunotherapy and dCRT remain unresolved.

There are three options for the timing of combining dCRT and immunotherapy: simultaneous dCRT and immunotherapy, immunomaintenance therapy after dCRT, and dCRT after induction of immunotherapy. It is unclear on which patients these three options will produce an effect and which option is the best choice. At present, we give priority to induction immunotherapy followed by dCRT for EC patients who are prone to esophageal perforation as well as hemorrhage. The results of the ImpactCRT study (76) suggest that induction chemotherapy plus camrelizumab followed by dCRT on inoperable LA-ESCC patients has a promising efficacy and favorable tolerance profile. This combination therapy strategy deserves to be scrutinized and should be validated in future larger clinical trials. For patients with EC who are thought to tolerate dCRT, most studies have favored simultaneous dCRT and immunotherapy or immunomaintenance therapy after dCRT. In terms of the dosage of radiotherapy, the ongoing KEYNOTE-975 (77) study is conducted to assess the effectiveness and safety of 50Gy and 60Gy when combined with immunotherapy (77), which is an good example for future studies. As for the target volume, given the dependence of immunotherapy on lymphocyte function, the majority of clinical studies on dCRT combined with immunotherapy for EC patients have opted for IFI. However, there are no clinical studies examining whether the choice of ENI or IFI will has an impact on patient outcomes on the basis of the combination of dCRT and immunotherapy, which is a topic worth discussing.

### 8.2 Definitive concurrent chemoradiotherapy combined with other therapies

Several studies have attempted to combine dCRT with other therapies to improve survival in patients with inoperable LA-ESCC, such as with antifungal drug, with oncolytic virus, with chemotherapy, and with targeted therapy (**Table 3**). However, the efficacy and safety of these combination therapies have not been reported, except for dcrt combined with targeted therapy. Over the past few years, there has been little progress in dCRT combined with targeted therapy for LA-ESCC. And one of the most critical points limiting its progress is that no suitable target has been found in the LA-ESCC patients. So far, epidermal growth factor receptor (EGFR) is the most commonly expressed target in patients with ESCC (78). However, previous SCOPE1 (79) study and RTOG0436 (80) study have shown that the combination of EGFR antibody and dCRT did not improve 1-year and 2-year OS or CRR of patients. And there seems to be a glimmer of hope in the NXCEL1311 study (81), whose interim results indicated that the combination of EGFR antibody and dCRT significantly improved the 3-month CRR and objective response rate in patients with LA-ESCC compared with dCRT alone. Nevertheless, we still need to wait for the 5-year results of this study to support whether patients will benefit from receiving targeted therapy in combination with dCRT. In the future, therapeutic targets for LA-ESCC deserve further investigation.

**Table 3:** Definitive concurrentchemoradiotherapy combined with other therapies for unresectable esophageal squamous cell carcinoma: ongoing trials.

Registration	Phase	Participants	Population	Study Cohort	Control Cohort	Combination therapy	Primary Endpoints	Secondary Endpoints
NCT04481100	II	38	T3-4N0M0, T1-4N+M0, stage II-IVA ESCC(AJCC 8th)	Itraconazole + Chemotherapy (NP) + Radiotherapy(NP)		Antifungal drug	ORR	LRFS, OS, AEs
NCT04391049	I	16	Unresectable GEJC/ESCC/EAC	OBP-301 + Chemotherapy (Carboplatin + Paclitaxel) + Radiotherapy(NP)		Oncolytic virus	Dose Limiting Toxicity	AEs, CCR, PFS, OS
NCT05512520 EC-CRT-003	II	126	Stage IVB ESCC(UICC 8th)	anti-PD1 + Chemotherapy (Fluoropyrimidine or Taxane-based platinum doublet chemotherapy) + Radiotherapy(40-50.4Gy/25-28F)	Chemotherapy (Fluoropyrimidine or Taxane-based platinum doublet chemotherapy) + Radiotherapy(40-50.4GY/25-28F)	Chemotherapy	PFS	OS, ORR, AEs
NCT02409186 NXCEL1311	III	200	Stage II-III cervical esophageal cancer, Unresectable esophageal cancer (AJCC 7th)	Nimotuzumab + Chemotherapy (Paclitaxel + Cisplatin) + Radiotherapy(59.4Gy/33F)	Placebo + Chemotherapy (Paclitaxel + Cisplatin) + Radiotherapy(59.4GY/33F)	Targeted Therapy	OS	

T, Tumor; N, regional lymph node; M, Metastasis; ESCC, esophageal squamous cell carcinoma; EAC, esophageal adenocarcinoma; GEJC, gastroesophageal junction cancer; AJCC, American Joint Committee on Cancer; UICC, Union for International Cancer Control; F, fraction; NR, not reported; ORR, objective response rate; LRFS, locoregional recurrence free survival; OS, overall survival; AEs, adverse events; CCR, clinical complete response; PFS, progression-free survival.

### 8.3 Patient selection and predictive markers

Considering the heterogeneity of LA-ESCC, selection of appropriate patients for comprehensive therapy is critical. However, we do not have an ideal biomarker to accurately screen appropriate patients for the application of dCRT in combination with other treatments. The most recognized molecular markers are PD-L1, microsatellite instability and tumor mutational load, but they are still some way from being ideal predictive markers. Various external factors, including sample quality, assay conditions, and cutoff values can influence the predictive accuracy of these markers (82–84).

In addition, there are a number of molecular biomarkers that have been reported to be valuable in screening appropriate patients, such as circulating tumor DNAs, expression molecular subtypes, dendritic cells, cytokines, and gut microbiota (85–92). Nevertheless, the utilization of these biomarkers in clinical practice remains limited due to high cost and low accuracy, and there is a lack of proof to support the notion that these biomarkers can forecast the effectiveness of dCRT in conjunction with other therapies for patients diagnosed with EC.

Deep learning models that utilize imaging have a promising capability to forecast the response to cancer treatment or prognosis (93, 94). However, enhancing the precision of forecasts requires the optimization of particular methods and factors (95–97). Data from clinical studies on LA-ESCC are gradually increasing, and more and more studies are beginning to apply PET/CT to assess patient treatment efficacy and to guide treatment decisions based on imaging response (35, 98, 99). There are even new PET imaging techniques that can detect tumor PD-L1, allowing noninvasive, real-time monitoring of changes in a patient’s PD-L1 status (100).

Given the heterogeneity of tumors, different EC patients need very different treatment plans. Tumor volume, location, radiotherapy dose, and chemotherapy regimen are all important factors affecting the efficacy of dCRT for EC patients, so we urgently need more accurate molecular biomarkers to select appropriate treatment plans for patients.

## 9 Conclusions

At present, dCRT is still the first choice of treatment for inoperable LA-ESCC. The preferred radiotherapy is IMRT, which uses IFI and a recommended dose of 50-50.4 Gy. Optional chemotherapy regimens include oxaliplatin plus fluorouracil, FOLFOX, and cisplatin plus fluorouracil. Immunotherapy combined with dCRT is a promising treatment for inoperable LA-ESCC, which is expected to improve long-term survival of patients. However, when dCRT is combined with immunotherapy, the optimal irradiation dose, segmentation scheme, radiotherapy technique, timing, sequence and duration for combining dCRT with immunotherapy, as well as the selection of chemotherapeutic and immunologic drugs need to be explored. In addition, we need to continue to explore the synergistic anti-tumor mechanisms of dCRT and immunotherapy and the molecular biomarkers that enable precise screening of appropriate patients.

## Author contributions

RX: Writing – original draft. QC: Writing – review & editing. TC: Writing – review & editing. HH: Writing – review & editing. CC: Supervision, Validation, Writing – review & editing.
